# GPTNeXt: Biomedical Image Classification Investigations

**DOI:** 10.3390/diagnostics16040581

**Published:** 2026-02-14

**Authors:** Fahad A. Alotaibi, Mehmet Said Nur Yagmahan, Khalid A. Alobaid, Mousa Jari, Omer Faruk Goktas, Mehmet Baygin, Turker Tuncer, Sengul Dogan

**Affiliations:** 1 College of Applied Computer Sciences (CACS), King Saud University, Riyadh 11543, Saudi Arabia; faalotaibi@ksu.edu.sa (F.A.A.); kalobaid@ksu.edu.sa (K.A.A.); maljari@ksu.edu.sa (M.J.); 2 Department of Computer Engineering, College of Engineering, Erzurum Technical University, Erzurum 25050, Turkey; mehmet.yagmahan@erzurum.edu.tr (M.S.N.Y.); mehmet.baygin@erzurum.edu.tr (M.B.); 3 Department of Electronics and Automation, Technical Sciences Vocational School, Ankara Yildirim Beyazit University, Ankara 06010, Turkey; ofgoktas@aybu.edu.tr; 4 Department of Digital Forensics Engineering, College of Technology, Firat University, Elazig 23119, Turkey; turkertuncer@firat.edu.tr

**Keywords:** GPTNeXt, deep feature engineering, biomedical image classification, deep learning, convolutional neural network

## Abstract

**Background/Objectives**: In the field of computer vision, prominent solutions often rely on transformers and convolutional neural networks (CNNs). Researchers frequently incorporate CNNs and transformers in developing image classification models. This study aims to introduce an innovative CNN model inspired by the Generative Pretrained Transformer (GPT) architecture and assess its image classification capabilities. **Methods**: This study utilized three distinct biomedical image datasets to evaluate the efficacy of the proposed GPTNeXt model. The datasets encompassed (i) Alzheimer’s disease (AD) magnetic resonance (MR) images, (ii) blood images, and (iii) lung cancer images. The choice of these datasets aimed to showcase the GPTNeXt model’s versatile classification performance. The GPTNeXt model and a deep feature engineering approach based on it were developed. In this deep feature engineering model, features were extracted from the global average pooling layer of GPTNeXt, and a novel deep feature extraction method was employed. This method extracted features from the entire image and generated nine fixed-size patches. To identify the most informative features, iterative neighborhood component analysis (INCA) was applied. The classification phase involved three shallow classifiers to produce classification results. **Results**: The GPTNeXt-based feature engineering model was applied to the three aforementioned biomedical image datasets, achieving classification accuracies exceeding 98% for all of them. **Conclusions**: This study demonstrates the high effectiveness of the proposed approach, as evidenced by the exceptional classification performance on the selected biomedical image datasets. Additionally, a lightweight CNN was introduced, showcasing outstanding classification performance.

## 1. Introduction

Biomedical image classification is a scientific field which comprises both pixel-wise analysis of images from medical imaging modalities and automatic detection and categorization of the corresponding features [1,2]. Its goal is to assist healthcare workers by examining images that are essential for diagnosing and treating diseases [3,4]. Techniques such as Magnetic Resonance Imaging (MRI) [5], Computed Tomography (CT) [6], Ultrasound [7], X-ray [8], and Positron Emission Tomography (PET) [9] generate fine-grained information from various body parts that are necessary for improved disease diagnoses, treatment planning and monitoring of the progression of diseases [10]. Biomedical image classification is widely used in diseases diagnosis, therapeutic outcome monitoring, and research/development [11].

Thanks to artificial intelligence and machine learning, the algorithms adopted for biomedical image classification have developed strikingly [12]. Given a rich set of visual features, such methods are well-suited for the identification of intricate patterns or fine-grained details in an image, often matching or even exceeding human-level performance and accuracy [13,14]. This results in improving the efficacy of diagnostic/therapeutic activities [15].

Specialist professionals operating in this area combine expertise from different domains that include computer science, artificial intelligence, image processing as well as medical and biological sciences [16]. This cross-disciplinary approach is essential to develop more sophisticated and accurate methods for medical image analysis. As a result, biomedical image classification has become an essential part of modern medicine in terms of improving and promoting the quality of care [2]. The three disease domains selected for this study—Alzheimer’s disease, blood cell abnormalities, and lung cancer—represent critical areas where automated image classification can significantly impact clinical outcomes. Alzheimer’s disease affects over 55 million people worldwide and is projected to triple by 2050; early detection through MRI analysis is crucial as it enables timely intervention during the mild cognitive impairment stage when treatments are most effective, yet subtle structural changes in brain images make early-stage differentiation particularly challenging for clinicians. Blood cell classification is fundamental to diagnosing hematological disorders, infections, and malignancies; however, manual microscopic examination is time-consuming, subjective, and requires specialized expertise, creating a bottleneck in clinical laboratories processing thousands of samples daily. Lung cancer remains the leading cause of cancer-related mortality globally, with five-year survival rates dramatically improving from 18% to over 60% when detected at early stages; histopathological image analysis is essential for accurate subtype classification (adenocarcinoma, squamous cell carcinoma, and neuroendocrine), which directly influences treatment selection. These three domains collectively represent diverse imaging modalities (MRI, microscopy, and histopathology), varying classification complexities (4–8 classes), and different spatial scales of discriminative features, making them ideal candidates for evaluating the generalizability of our proposed approach.

In this paper, we present the GPTNeXt model as a new architecture for a CNN based on the GPT framework in order to demonstrate that efficient CNN architectures can still achieve competitive performance compared with transformer-based approaches. GPTNeXt is trained to cope with the difficulties of medical images, providing an interpretable and meaningful approach towards classification problems in biomedical applications.

Some recent studies for the three diseases selected for this article are presented below.

Kaplan et al. [17] proposed ExHiF, an approach for AD detection using exemplar histogram-based features extracted from CT and MR images. The method demonstrated the potential for effective AD classification by leveraging texture and shape information from medical images. By combining features from different modalities, ExHiF offered a robust approach that could contribute to improving AD diagnosis accuracy. They achieved an accuracy rate of 100.0% with tenfold cross-validation. Lanjewar et al. [18] developed a framework that combines CNN with kNN for AD detection using MRI images. They used 6400 MRI images with four classes. They attained an accuracy value of 99.58% with 80:20 ratios. de Mendonca [19] investigated the use of graph kernel SVM for Alzheimer’s disease classification. They employed 3D texture features extracted from 474 MR images. They obtained an accuracy of 83.80% with tenfold cross-validation. Shamrat et al. [20] introduced AlzheimerNet, a deep learning-based model for classifying different stages of AD. Their approach leverages the power of deep neural networks to capture complex patterns and variations in brain scans, facilitating the accurate classification of AD stages. They calculated accuracy was 98.67% with a ratio of 60:20:20 using 23,508 MR images. Arafa et al. [21] suggested a scheme for the early diagnosis of AD based on MR images. Their research emphasized the significance of early detection and demonstrates the application of deep learning models to that end. They achieved a high accuracy (99.99%) for the classification of AD stages with a 70:30 ratio. Marwa et al. [22] proposed a method based on MRI for the accurate diagnosis of AD. In their study, they outlined the contribution of deep learning to improving precision (99.68%) and reliability for AD diagnosis. The work in [23] used deep features for white blood cell detection and automatic classification based on the CNNs proposed by Yentrapragada. The deep feature extraction of the model makes it capable of distinguishing various types of white blood cells accurately. They achieved an accuracy of 97.00% with an 80:20 ratio as well. Ahmad et al. [24] also proposed deep features optimization with entropy control for white blood cell classification. Their approach used entropy as a guiding principle of feature selection. Their work emphasized the need for feature selection in DL-based models for medical imaging analysis. They obtained a 99.90% accuracy with 80:20 ratios. Manescu et al. [25] proposed a technology for the diagnosis of acute promyelocytic leukemia in peripheral blood and bone marrow based on annotation-free deep learning. It is worth noticing that their study was annotation-free by allowing the neural network itself to detect disease markers. The accuracy ratio was 99.00% at threefold cross-validation. In Leng et al. [26], a detection transformer based on deep learning was applied for the detection of peripheral blood leukocytes. Their tracking methodology improved the precision and speed of detecting leukocytes in blood samples. They achieved a recall of 94.30%. The introduction of a detection transformer shed light on the applicability of transformer-based approaches in medical image analysis. Rahman et al. [27] coped with the multiclass blood cancer classification task by using a deep CNN model with optimized features. They aimed to improve the classification accuracy of diverse blood cancer types by presenting a combined solution for cancer diagnosis. A tenfold cross-validation resulted in an accuracy rate of 99.84%. Hamed et al. [28] introduced an approach for lung cancer histopathology classification by combining CNN and the LightGBM algorithm. Their hybrid model enhanced the accuracy of histopathological image analysis, demonstrating the synergy between deep learning and gradient boosting methods. They achieved an accuracy value of 99.60% with 60:40 ratios. Recent advances in attention-based CNN architectures have shown promising results in medical image classification. Huang et al. [29] proposed FABNet, which combines fusion attention blocks with transfer learning for laryngeal cancer tumor grading in P63 IHC histopathology images, achieving improved accuracy through clinical a priori experience-guided model transfer and non-uniform sparse representation. Zhou et al. [30] introduced LPCANet, employing position attention and channel attention mechanisms based on ResNet50 to capture global and channel relationships in laryngeal cancer histopathological images, achieving an 83.15% accuracy with enhanced interpretability through Grad-CAM visualization. Luo et al. [31] developed DCA-DAFFNet, utilizing deformable convolution-guided attention blocks and deep adaptive feature fusion to adaptively represent nuclei with variable morphologies, achieving a 90.78% accuracy for laryngeal tumor grading. Raza et al. [32] proposed Lung-EffNet, a deep learning model based on the EfficientNet architecture for lung cancer classification using CT scan images. Their approach leveraged the scalability and efficiency of EfficientNet to achieve improved accuracy in lung cancer detection. The obtained accuracy was 99.10% for a ratio of 80:20. Malik et al. [33] introduced CDC_Net, which is a multiclassification CNN model for identifying multiple lung-related diseases along with lung cancer through chest X-ray imagery. The model demonstrated the ability to find several pathological opportunities for lung health screening using a comprehensive analysis procedure. They achieved an average accuracy of 99.39% using tenfold cross-validation. Their study responded to the call for comprehensive diagnostic practices in medicine. Quasar et al. [34] investigated ensemble procedures to enhance lung cancer detection and classification with CT scan images. They achieved an accuracy of 98.00% with a ratio of 70:20:10 for all three categories. Their research highlighted the potential of combining multiple models to enhance diagnostic accuracy and reduce false positives. Beyond CNN-based attention mechanisms, transformer-based architectures have recently demonstrated remarkable performance in medical image classification. Huang et al. [35] proposed ViT-AMC, an end-to-end network that integrates Vision Transformer (ViT) blocks with attention mechanism-integrated convolution (AMC) blocks through adaptive model fusion and multiobjective optimization, addressing the trade-off between interpretability and inductive bias capability for laryngeal tumor grading. The same research group further developed MamlFormer [36], a transformer network incorporating manifold adversarial multimodal learning to effectively fuse high- and low-magnification histopathological images while introducing pathologists’ prior experience for improved grading performance and interpretability. For breast cancer diagnosis, Huang et al. [37] presented a breast tumor grading network based on adaptive fusion and microscopic imaging, combining ViT and CNN blocks with integrated attention to achieve a 95.14% accuracy, with visualization maps consistent with pathologists’ regions of interest. While these transformer-based methods achieve strong performance with enhanced interpretability, they typically involve complex multi-branch architectures, sophisticated fusion mechanisms, and substantial computational requirements. Our proposed GPTNeXt takes a fundamentally different approach: rather than combining separate CNN and transformer branches, we incorporate transformer-inspired design principles (Pre-LN normalization, GELU activation, patchify stems, and block-wise hierarchical design) directly into a unified lightweight convolutional framework. This design philosophy achieves competitive classification performance with only 7.4 M parameters—significantly fewer than typical ViT-CNN hybrid architectures—while our exemplar-based feature engineering provides an alternative pathway to multi-scale feature extraction without requiring explicit attention mechanisms.

Outside disease specific applications, the recent progress of biomedical deep learning has shown how crucial architectural considerations, as well as feature robustness and severe evaluation protocols, are across various medical imaging modalities. For instance, Iqbal et al. [38] proposed a deep learning approach to the morphological classification of the human sperm head while pointing out obstacles, such as the scarcity of training samples and variety of morphologies, that are commonly found in medical imaging. Similarly, Iqbal et al. [39] proposed a deep CNN-based automated system for the detection of gastrointestinal abnormalities in endoscopic images, and highlighted the importance of multi-scale feature extraction for effective training as well as strong evaluation strategies that reflect the clinical deployment process. Together, these works highlight several important factors in the domain of biomedical image classification: (i) lightweight architectures that are able to run in resource-limited clinics; (ii) robust feature extraction techniques that can generalize across imaging modalities and acquisition protocols; (iii) thorough statistical validation, such as confidence intervals and cross-validation; and interpretability tools to gain clinically relevant insights. These issues are dealt with in our GPTNeXt framework through effective architectural design and multi-scale patch-based feature construction, as well as full statistical analysis.

Although these studies have demonstrated high performance in terms of classification accuracy, several common limitations remain, which motivated our method. First, most state-of-the art methods are built using heavy deep learning models (e.g., VGG16, ResNet-50 and InceptionResNetV2) with millions of parameters, making such methods computationally expensive and difficult to deploy in a resource-limited clinical setting. Second, the majority of methods use end-to-end softmax classification to predict the disease category, yielding little interpretability in terms of determining what parts of an image contribute to this decision, which is essential for clinical acceptability. Third, single-scale feature extraction used in most studies may overlook discriminative patterns that exist at different spatial granularities, e.g., Alzheimer’s disease exhibits a global brain atrophy pattern as well as localized hippocampal change. Fourth, the evaluation setups are widely different from each other throughout studies, such as hold-out ratios (from 60:40 to 84:16) and cross-validation folds (3 up to 10), making it hard to compare performances directly. Fifth, some high-precision claims are made without strict statistical validation (e.g., confidence interval or significance testing), bringing into question the reproducibility of the results. Taken together, these limitations emphasize the importance of an efficient multi-scale feature extraction technique with strong statistical validation, which is the essence of our proposed GPTNeXt-based framework.

To provide a clear overview of our research pipeline, (i) we first introduce GPTNeXt, a lightweight CNN architecture (7.4 M parameters) incorporating transformer-inspired design principles; (ii) we train GPTNeXt on three biomedical image datasets; (iii) we develop an exemplar-based deep feature engineering model that extracts multi-scale features from both the full image and nine overlapping patches using the pretrained GPTNeXt; (iv) we apply INCA-based feature selection to identify the most discriminative features; and (v) we evaluate the classification performance using three shallow classifiers (kNN, SVM, and LDA) with 10-fold cross-validation. This two-stage approach combines the representation learning capability of deep networks with the interpretability and efficiency of traditional machine learning classifiers.

### 1.1. Motivation and Our Model

Recently, the paradigm of LLMs has become widely prevalent and has shaken up the entire technology industry [40]. For instance, chat applications (such as ChatGPT and Bard) have harnessed LLMs to simplify the lives of billions of users [41]. These applications help make work go more smoothly and lead to increased productivity in both personal and professional life [42]. At the heart of these advances are transformers that, as the building blocks of LLMs, constitute a changing front line in the field of machine learning [43].

This study focuses on biomedical image classification. We propose a novel CNN model inspired by transformer design principles, specifically adapting structural elements from the GPT architecture into an efficient convolutional framework. This pursuit culminates in the creation of GPTNeXt. It is important to clarify that the term “GPT-inspired” in our work refers to design principles rather than direct architectural components. Unlike transformer-based models, GPTNeXt does not incorporate self-attention or token mixing mechanisms. Instead, we adopt the following design philosophies from the GPT paradigm: (i) block-wise hierarchical design with repeated modular blocks, (ii) Pre-LN normalization placement where layer normalization is applied at the beginning of each block, (iii) simplified and scalable architecture that can be extended through repetition factors, and (iv) a GELU activation function instead of the conventional ReLU. These design choices align GPTNeXt with modern CNN architectures, such as ConvNeXt, while maintaining a lightweight footprint suitable for biomedical applications. GPTNeXt demonstrates superior classification accuracy across three publicly accessible biomedical image datasets having undergone critical appraisals. For us, the ideal is to develop a lightweight CNN model—the lightness of GPTNeXt embodies that goal, with a modest 7.4 million learnable parameters—and following a simple architectural model.

Additionally, our work tries to plant the seeds of the following two aspects: deep learning and engineering in future innovation. It is with GPTNeXt that we establish a new model of deep learning, and in doing so, give birth to a new technique for deep feature engineering.

This work has two main components: (i) the proposed GPTNeXt architecture and (ii) an exemplar-based deep feature engineering pipeline built on pretrained GPTNeXt features, followed by INCA-based feature selection and classification using kNN, SVM, and LDA.

### 1.2. Novelties and Contributions

The main contributions of this work are threefold:

- Architectural Innovation: We propose GPTNeXt, a lightweight CNN (7.4 M parameters) that incorporates transformer-inspired design principles including a patchify stem, Pre-LN normalization, GELU activation, and dual shortcut connections within an efficient convolutional framework.
- Feature Engineering Framework: We introduce an exemplar-based deep feature engineering approach that extracts multi-scale representations through fixed-size patch decomposition, combined with INCA-based feature selection for dimensionality reduction.
- Empirical Validation: We demonstrate consistent classification performance exceeding 98% accuracy across three diverse biomedical imaging datasets, with comprehensive statistical validation including confidence intervals and significance testing.

## 2. Materials and Methods

### 2.1. Material

In order to demonstrate the general classification abilities of our proposed GPTNeXt model, we experimented with datasets comprising three different dataset types, all belonging to biomedical image datasets. These were downloaded from Kaggle, and we present descriptions of each dataset in the following sections.

#### 2.1.1. Alzheimer’s MR Image Dataset (AD)

The augmented Alzheimer’s MR image dataset [44] was the first dataset used in this study. The dataset has been downloaded from the following link: https://www.kaggle.com/datasets/uraninjo/augmented-alzheimer-mri-dataset-v2 (accessed on 21 January 2025). It consists of images related to four different classes. We used the augmented images for training and tested on the original images.

Important Note on Data Separation: The augmented Alzheimer’s MRI dataset maintains strict separation between training and test data. The augmented images used for training were generated from a distinct source dataset, while the test set contains only original, non-augmented images. This design ensures that no data leakage occurs between training and test sets, as confirmed by the dataset documentation, which states that ‘Originals could be used for validation or test dataset.’ We verified this separation by confirming that no test image or its augmented variant exists in the training set.

#### 2.1.2. Blood Image Cell Dataset (Blood)

The second dataset [45,46] used in the current study is the blood image cell dataset, with eight classes. A total of 17,092 images are in this dataset. We downloaded this dataset from the following link: https://www.kaggle.com/datasets/unclesamulus/blood-cells-image-dataset (accessed on 18 January 2025). 

#### 2.1.3. Lung Cancer Image Dataset

The third dataset is the lung cancer image dataset, made up of three categories of lung cancer images [47]. We obtained this dataset from Kaggle using: https://www.kaggle.com/datasets/bhaveshmisra/lung-cancer-images12000-imagesmostly (accessed on 18 January 2025). It contains 12,000 training images and 2,997 test images in total.

The three datasets have train and test folders. The essential features of these datasets, as well as their statistics, are summarized in Table 1.

These datasets collectively serve as the foundation for our experiments and analyses. The class distribution analysis reveals varying degrees of imbalance across datasets. The Lung Cancer dataset exhibits perfect balance (1:1:1 ratio). The AD dataset shows moderate imbalance, with the moderate dementia class being underrepresented (16.2% of total samples). The Blood Cell dataset presents the highest imbalance, with Basophil and Lymphocyte classes comprising only 7.1% each compared to Neutrophil at 19.5%. To address potential bias from class imbalance, we employed: (i) stratified sampling during cross-validation to maintain class proportions, (ii) Unweighted Average Recall (UAR) and Unweighted Average Precision (UAP) metrics that treat all classes equally regardless of size; and (iii) per-class performance analysis through confusion matrices.

It should be noted that the datasets used in this study were obtained from publicly available Kaggle repositories, which do not provide patient-level metadata. Consequently, patient-level separation between training and test sets cannot be explicitly verified. This is a common limitation when utilizing public benchmark datasets, as individual patient identifiers are typically anonymized or unavailable. For the Alzheimer’s MRI dataset, the data providers explicitly separated augmented images (for training) from original images (for testing), which provides an alternative form of data separation that prevents direct data leakage. For the Blood Cell and Lung Cancer datasets, the predefined train–test splits provided by the dataset curators were adopted. We acknowledge that future studies using clinical datasets with patient-level annotations would provide stronger validation of generalization performance.

Regarding acquisition variability, the Alzheimer’s MRI dataset contains images acquired from multiple clinical sources with varying scanner parameters, representing realistic heterogeneity encountered in clinical practice. The Blood Cell dataset comprises microscopic images captured under standardized laboratory conditions with consistent staining protocols. The Lung Cancer histopathology dataset includes images from different tissue preparations, introducing natural variability in staining intensity and tissue morphology. This diversity across datasets enables evaluation of our method’s robustness to acquisition-related variations.

Regarding preprocessing, we deliberately employed minimal preprocessing to evaluate the robustness of our proposed method on raw image data. Specifically, no additional normalization, histogram equalization, or contrast enhancement was applied beyond the default processing inherent to the GPTNeXt architecture (standard input scaling to [0, 1] range). Images were resized to 224 × 224 pixels to match the network input requirements using bilinear interpolation. Importantly, no data augmentation techniques (rotation, flipping, color jittering, etc.) were applied during our feature engineering pipeline, as the Alzheimer’s dataset already provided pre-augmented training images, and we aimed to assess the model’s performance without artificial data expansion for the other datasets. This minimal preprocessing approach ensures reproducibility and demonstrates that our method achieves high classification performance without relying on extensive preprocessing pipelines.

### 2.2. GPTNeXt

In this paper, we propose a new type of CNN model, referred as GPTNeXt, with the aim of providing an extremely high accuracy ratio and a model that can be reused in embedded systems and simplified. This takes advantage of the fact that the GPT model is quite simplistic. The basis of GPTNeXt is the efficient block called the GPTNeXt block, including four core modules: (i) the stem block; (ii) the GPTNeXt block; (ii) downsampling blocks; (iv) output blocks.

**Figure 1:**
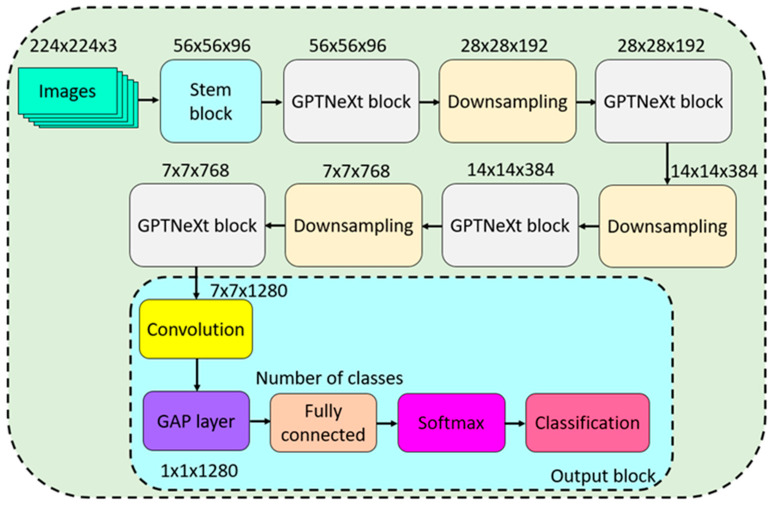
Graphical explanation of the proposed GPTNeXt deep learning model. GAP: global average pooling.

The structure of GPTNeXt is built by the combination of these four blocks. In order to help visualize this model, we depict a graphical view of GPTNeXt in Figure 1, and we describe its components in the following sections:

Stem block: The stem block is the first component in GPTNeXt and uses patchify. Inside this block, we applied a convolution operator with the following features: 96 filters, a filter size of 4 × 4 and stride value of 4 × 4. This convolution operation converts a 224 × 224 × 3 input image into a 56 × 56 × 96 tensor. Then batch normalization and GELU functions are used.

**Figure 2:**
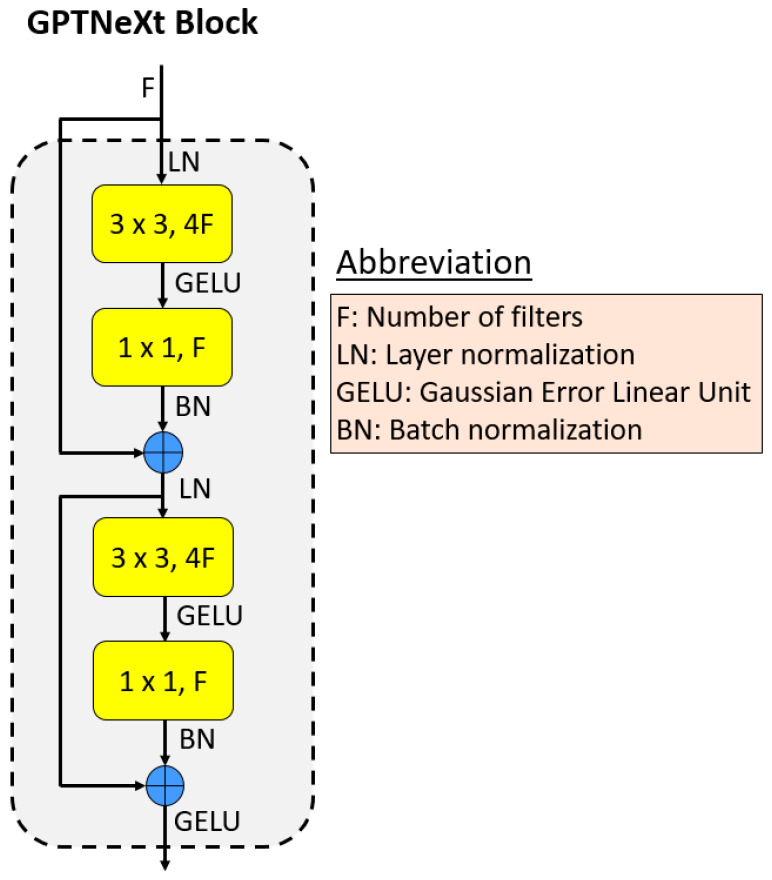
Graphical depiction of the GPTNeXt block.

GPTNeXt block: The central part of GPT is the GPTNeXt block, which acts as an effective feature map producer. Its graphical shape is shown in Figure 2.

The codes of proposed GPTNeXt model are given in Table A1 and Table A2. Inside the GPTNeXt block, we use layer normalization, convolution and GELU batch normalization. Further, two shortcuts are incorporated to address the vanishing gradient problem. This combination with normalization, convolution and activation functions is new. In particular, the first convolution consists of a depth-wise convolution (3 × 3 convolution) followed by a point-wise convolution. An architecture with an inverted bottleneck structure is adopted to produce feature maps.

Downsampling block: A downsampling block based on patchify is adopted to downsample the feature map. This block is composed of three operations, namely, convolution, layer normalization and GELU. No pooling operators are used to prevent routing problems. The convolutions in this block are designed to reduce the feature map and increase the number of filters. A grouped convolution operator with a 2 × 2 filter size, a stride of 2 × 2 and two filters per group are then used. These characteristics highlight the use of patchify in terms of reducing the feature map.

Output block: The final component of GPTNeXt is the output block. Initially, a pixel-wise convolution is employed to increase the number of filters from 768 to 1280. This convolution operation is followed by a combination of convolution, layer normalization, GELU, and batch normalization. Subsequently, a global average pooling operation is applied to generate a one-dimensional feature map. The classification outcome is obtained through the application of fully connected and softmax operations.

Moreover, we demonstrate the configuration of the proposed GPT using a symbolic mathematic model. F denotes the number of filters and R denotes the repetition number. The mathematical definition is given below.

(1) $$GPTNeXt:F=\left(96,\;192,\;384,\;768,\;1280\right),\;R=(1,\;1,\;1,\;1,\;1)$$

To clarify the presented GPTNeXt, the transition table of this model is illustrated in Table 2.

As indicated in Table 2, our proposed model employs a varying number of filters, including 96, 192, 384, 768, and 1280. To transition from the first to the fourth set of filters, we incorporate four GPTNeXt blocks. Notably, we employ pixel-wise convolution approximation to augment the number of filters from 1280 to 768. It is important to note that this structure does not follow a repetitive sequence of construction. Accordingly, our model design is deliberately simple so to act as a base network. The possibility of building larger GPTNeXt-based systems with higher repetitions is left open for future investigation. GPTNeXt shares architectural similarities with ConvNeXt [48], a modernized CNN that also incorporates design principles from transformers. However, several key differences distinguish our approach. Table 3 summarizes the comparison between GPTNeXt and ConvNeXt.

As shown in Table 3, GPTNeXt is designed as a more compact architecture with approximately 74% fewer parameters than ConvNeXt-Tiny, making it particularly suitable for resource-constrained biomedical imaging applications.

While GPTNeXt shares certain design principles with ConvNeXt [48], several critical architectural choices distinguish our approach and make it particularly suitable for biomedical image classification:

- Lightweight Design (7.4 M vs. 28.6 M parameters): GPTNeXt achieves a 74% parameter reduction compared to ConvNeXt-Tiny, enabling deployment in resource-constrained clinical environments and embedded systems commonly used in point-of-care settings. This reduction is achieved without sacrificing performance, where GPTNeXt achieves comparable or superior accuracy to ConvNeXt-T across all three biomedical datasets.
- Grouped Downsampling with Smaller Kernels (3 × 3 vs. 7 × 7): Unlike ConvNeXt’s 7 × 7 depth-wise convolutions, GPTNeXt employs 3 × 3 grouped convolutions with dual shortcut connections. This design choice: (i) reduces computational cost (1.8 GFLOPs vs. 4.5 GFLOPs) while maintaining receptive field coverage through stacked layers; (ii) better preserves fine-grained details critical for biomedical imaging, such as cellular morphology in blood cell images, tissue boundaries in histopathology, and subtle atrophy patterns in brain MRI; and (iii) provides implicit regularization through parameter sharing, which is beneficial for limited-size medical datasets.
- Dual Shortcut Connections: GPTNeXt introduces two residual connections within each block (as shown in Figure 2) compared to ConvNeXt’s single shortcut connection. This architectural choice enhances gradient flow during training and enables more stable convergence on limited biomedical datasets where overfitting is a primary concern. The dual shortcut s contribute +0.93% accuracy improvement over single shortcuts.
- Biomedical-Specific Design Rationale: Our architectural decisions are motivated by unique characteristics of biomedical image classification: (i) medical images often contain subtle, localized abnormalities (e.g., early-stage tumor markers and cellular morphology variations) requiring fine-grained feature extraction; (ii) limited dataset sizes compared to natural image datasets (e.g., ImageNet) benefit from lightweight architectures with inherent regularization properties; (iii) clinical deployment scenarios prioritize inference efficiency (4.5 ms per image) and model compactness (28.2 MB) for integration into existing hospital information systems; and (iv) multi-scale feature extraction (full image + patches) is essential for capturing both global context and local pathological patterns.
- Feature Engineering Compatibility: Unlike ConvNeXt’s end-to-end classification paradigm, GPTNeXt’s architecture is specifically designed to generate discriminative features at the global average pooling layer, enabling effective integration with our patch-based exemplar feature engineering pipeline. The architectural simplicity (straight-forward design without complex attention mechanisms) facilitates feature extraction stability across different image patches, as evidenced by the consistent performance improvements, when patch-based features are incorporated.

These architectural differentiations collectively enable GPTNeXt to achieve state-of-the-art classification performance while maintaining computational efficiency and generalizability across diverse biomedical imaging modalities (MRI, microscopy, and histopathology).

### 2.3. GPTNeXt-Based Exemplar Deep Feature Engineering

In this work, we investigate our CNN architecture on top of GPTNeXt to improve the classification accuracy. We propose the other deep feature engineering model on top of the GPTNeXt architecture that utilizes the pretrained GPTNeXt. The Exemplar Deep Feature Engineering Model based on GPTNeXt consists of three main stages. (i) Exemplar Deep Feature Extraction for the Pretrained GPTNeXt: Global Average Pooling (GAP) is performed to extract deep features from the pretrained GPTNeXt. (ii) INCA-Based Feature Selection: We perform feature selection using an INCA-based method, aiming to select and keep the most informative features. Classification for kNN, SVM and LDA Classifiers: In this step we classify with the k-Nearest Neighbors (kNN), Support Vector Machine (SVM), Linear Discriminant Analysis (LDA).

**Figure 3:**
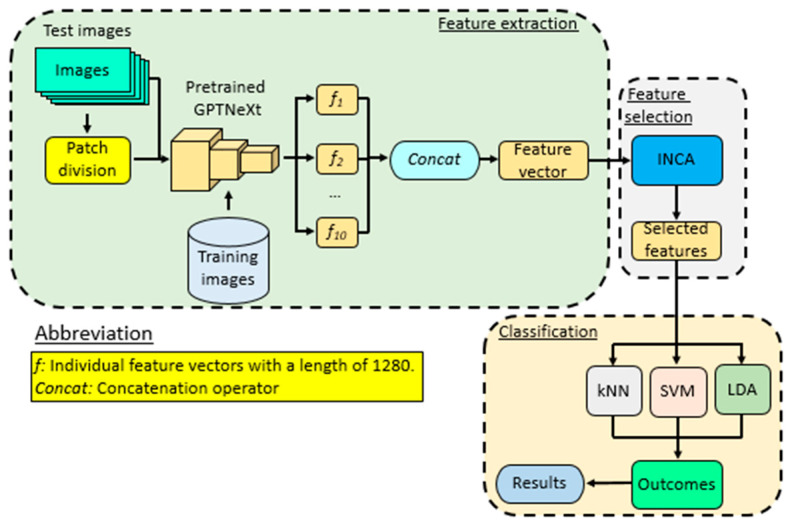
Overview of the proposed GPTNeXt-based deep feature engineering model.

A schematic of this deep feature engineering model is shown in Figure 3.

In this method, we divide the input image into nine overlapping fixed-size patches (112 × 112). Feature extraction is implemented using the pretrained GPTNeXt model. We use the Global Average Pooling (GAP) layer of the pretrained GPTNeXt to obtain features from both whole images and nine patches. Then, we employ 10 (=1 + 9) different feature vectors with length of 1280 (Figure 2 and Table 1). The ten feature vectors are concatenated to one, and the final feature vector has a length 12,800 (=1280 × 10). The proposed exemplar-based feature extraction employs nine overlapping patches of 112 × 112 pixels with a stride of 56 pixels. This design is motivated by several theoretical considerations:

- First, the patch size of 112 × 112 (half of the input resolution) ensures that each patch captures sufficient contextual information while enabling localized feature extraction. This multi-scale approach is analogous to spatial pyramid pooling, which has been theoretically shown to improve translation invariance and enable the capture of features at multiple granularities.
- Second, the overlapping stride of 56 pixels (50% overlap) ensures that discriminative features near patch boundaries are captured in multiple patches, reducing information loss at boundary regions. The resulting nine patches provide systematic coverage of the spatial domain: four corner regions, four edge–center regions, and one central region.
- Third, combining features from the full image with patch-level features creates a hierarchical representation that captures both global context (disease-level patterns) and local details (cellular or structural abnormalities). This dual-scale approach is particularly beneficial for biomedical images where diagnostic information may be distributed across different spatial scales.

The patch-based feature extraction strategy is particularly well-suited for histopathological image analysis due to the inherent characteristics of tissue specimens. Lung cancer histopathology images exhibit significant tissue heterogeneity, where discriminative features, such as cellular morphology, glandular formations in adenocarcinoma, keratin pearls in squamous cell carcinoma, and neuroendocrine rosettes in neuroendocrine tumors, may appear in localized regions rather than being uniformly distributed across the entire tissue section. Similarly, blood cell images contain multiple cells with varying morphological characteristics within a single field of view, and Alzheimer’s MRI scans present both global brain atrophy patterns and localized hippocampal changes. By extracting features from nine overlapping patches in addition to the full image, our method captures both global tissue/organ architecture and localized morphological patterns that are critical for accurate classification across all three biomedical domains.

To identify the most distinctive feature vector, we employ the INCA feature selector. The selected feature vector generated by INCA is subsequently subjected to classification using k-Nearest Neighbor (kNN), Support Vector Machine (SVM), and Linear Discriminant Analysis (LDA) classifiers, yielding the final results. To provide a clearer understanding of this model, we outline the steps below.

Step 1: Train the proposed GPTNeXt by deploying training images and obtain the pretrained GPTNeXt.

Step 2: “Load each test image and resize it to 224 × 224 × 3.

Step 3: Create overlapped fixed-sized patches. Herein, the size of the patch is determined as 112 × 112 × 3 and the stride is selected as 56.

(2) $$patch_{k}\left(i,j,:\right)=Im\left(w+i-1,h+j-1,:\right),\;i\in \left\{1,2,\ldots ,112\right\},\;\;\;\;\;j\in \left\{1,\;2,\ldots ,\;112\right\},w\in \left\{1,\;57,\;113\right\},\;h\in \left\{1,\;57,\;113\right\},\;k\in \left\{1,\;2,\ldots ,9\right\},\;$$

Herein, $patch_{k}$ implies the kth fixed-sized patch with a size of 112 × 112 × 3 and Im defines image.

Step 4: Generate individual feature vectors by deploying the GAP layer of the GPTNeXt.

(3) $$f_{1}=GPTNeXt(Im,GAP)$$

(4) $$f_{k+1}=GPTNeXt(patch_{k},GAP)$$

where f denotes the individual feature vector with a length of 1280 and GPTNeXt(.,.) is the pretrained CNN-based feature extraction function, which takes two parameters, the input image and the used layer, to get features.

Step 5: Create the ultimate feature vector by concatenating the generated individual feature vectors.

(5) $$F\left(r+1280\times \left(t-1\right)\right)=f_{t}\left(r\right),\;r\in \left\{1,\;2,\ldots ,\;1280\right\},t\in \left\{1,\;2,\ldots ,10\right\}\;$$

Here, F represents the ultimate feature vector with a length of 12,800 (=1280 × 10).

Step 6: Repeat steps 2–5 until the number of test images is reached, and create the feature matrix (X). The size of X is equal to NTI×12800, where NTI is number of test images.

The preceding six steps described above constitute the feature extraction phase of the GPTNeXt-based deep feature engineering model. Through this phase, we create the feature matrix. In the subsequent stage, our focus shifts to the elimination of redundant features. To achieve this, we implement the INCA feature selection function [51].

The INCA is an enriched version of the NCA [52] feature selection function. We use INCA to select the most discriminative features. INCA is a parametric feature selector with two parameters: (i) iteration range and (ii) a function computing the misclassification rate. Details on our use of the INCA-based automatic feature selection process are provided below.

**Figure 4:**
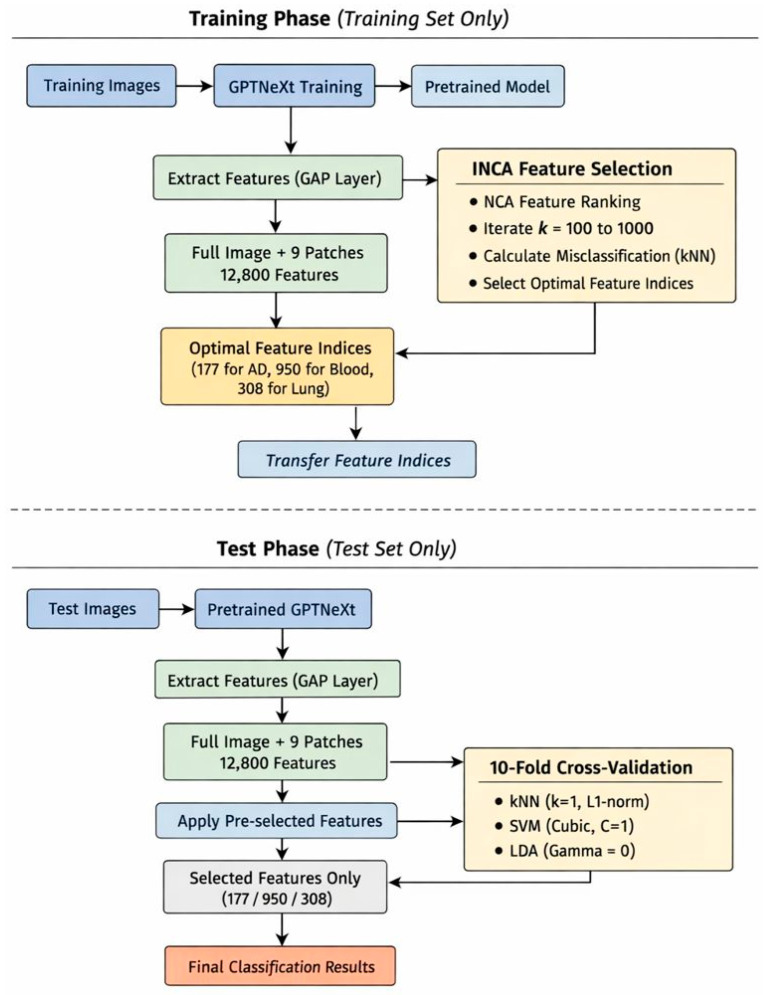
Experimental protocol demonstrating strict train–test separation in the GPTNeXt-based feature engineering pipeline. The INCA feature selection is performed exclusively on the training set (blue region), and the resulting feature indices are transferred to the test set (orange region) without any backward information flow. This design prevents data leakage and ensures statistically valid evaluation through 0-fold cross-validation was performed on the training set, and the final performance was reported on the independent test set.

Important Note on Train–Test Separation: It is crucial to emphasize that the INCA-based feature selection (steps 7–10) is performed exclusively on the training set. The feature indices selected through this process are then transferred to the test set for final evaluation. This strict separation ensures that no information from the test set influences the feature selection process, thereby preventing any potential data leakage. The complete workflow is illustrated in Figure 4.

Step 7: Apply the NCA to the feature matrix generated in the feature extraction phase and compute qualified indices of the created 12,800 features.

(6) id=NCA(X,y)

Here, id defines the qualified indices and y implies the actual outcomes.

Step 8: Choose the top features by using iteration and the generated indices.

(7) $$s_{a-iv+1}\left(q,w\right)=X\left(q,id\left(w\right)\right),\;a\in \left\{iv,iv+1,\ldots ,fv\right\},\;\;w\in \left\{1,\;2,\ldots ,a\right\},\;q\in \left\{1,\;2,\ldots ,NTI\right\}\;$$

where s is the selected features, iv is the initial value of the iteration and the minimum number of features, and fv is the final value of the iteration and the maximum number of features. Herein, the range is determined from 100 to 1000. Thus, 901 selected feature vectors have been created.

Step 9: Compute the misclassification ratios of the selected feature vectors.

(8) $$mcv(a-iv+1)=\gamma \left(s_{a-iv+1},y\right)$$

Herein, mcv defines the misclassification value and γ(.,.) is the misclassification value calculation function. In this research, we have used the kNN classifier [53] as the misclassification value calculation function.

Step 10: Choose the best feature vectors among to the selected feature vectors by deploying the computed misclassification values.

(9) $$\left[mini,\;ind\right]=min\;(mcv)$$

(10) $$sfv=s_{ind+iv-1}$$

where mini is the minimum misclassification value, ind is the index of the minimum misclassification value and sfv is the selected feature vector by INCA.

We computed the minimum misclassification rate and identified the optimal selected feature vector as the one corresponding to the index of the minimum misclassification value.

The final phase of the proposed deep feature engineering model involves classification. In this classification phase, we employed three shallow classifiers: k-Nearest Neighbors (kNN) [53], Support Vector Machine (SVM) [54], and Linear Discriminant Analysis (LDA) [55]. The selection of these classifiers was determined using the MATLAB2023b classification learner tool, which indicated their superior classification performance. Hence, these three classifiers were chosen for the classification step of the GPTNeXt-based model, as detailed below.

Step 11: Classify the selected feature vector by deploying kNN, SVM and LDA classifiers.

(11) $${out}_{1}=kNN\left(sfv,y\right)\;\;{out}_{2}=SVM\left(sfv,y\right)\;{out}_{3}=LDA(sfv,y)$$

where ${out}_{1}$,$\;{out}_{2}$ and ${out}_{3}$ are the outcomes generated by the used classifiers.

## 3. Experiments

In order to demonstrate the effectiveness of our proposed GPTNeXt and deep feature engineering method, we tested it on three datasets, as described in Section 2. The GPTNeXt and the GPTNeXt-based exemplar deep feature engineering models were developed by using MATLAB (version 2023b). The development of GPTNeXt has been made by using the deep learning model designer kit, which includes 73 operations and 80 connections. The generation process is quite simple. We then fine-tuned these models with the training set under the following settings:

GPTNeXt:

Solver: Stochastic Gradient Descent with Momentum (sgdm);
Initial Learning Rate: 0.01;
Maximum Epoch: 100;
Mini Batch Size: 32;
Training and Validation Ratio: 70:30.

Following the acquisition of the pretrained GPTNeXt, we proceeded to formulate the GPTNeXt-based exemplar deep feature extraction model using MATLAB codes. The components integrated into this proposed deep feature engineering model encompass: (i) GPTNeXt, (ii) Patch Divider, (iii) INCA, (iv) kNN, (v) SVM and (vi) LDA.

We generated the classifier codes for kNN, SVM, and LDA using the MATLAB classification learner tool. The attributes and configurations of these functions are outlined below:

For GPTNeXt-based Feature Engineering:

Pretrained GPTNeXt: GAP layer utilized to extract 1280 features;
Patch Divider: Patch size: 112 × 112, Stride: 56, and Number of created patches: 9;
INCA: Range of iteration: from 100 to 1000, Misclassification rate calculator: kNN, Solver: Stochastic Gradient Descent (sgd), and Number of iterations of NCA: half of the number of images;
kNN: k: 1, Distance metric: L1-norm, Voting: none, Validation: 10-fold cross-validation;
SVM: Kernel: cubic polynomial kernel, C: 1, Coding: one-vs-one, and Validation: 10-fold cross-validation;
LDA: Gamma: zero, Filling coefficients: off, and Validation: 10-fold cross-validation.

**Figure 5:**
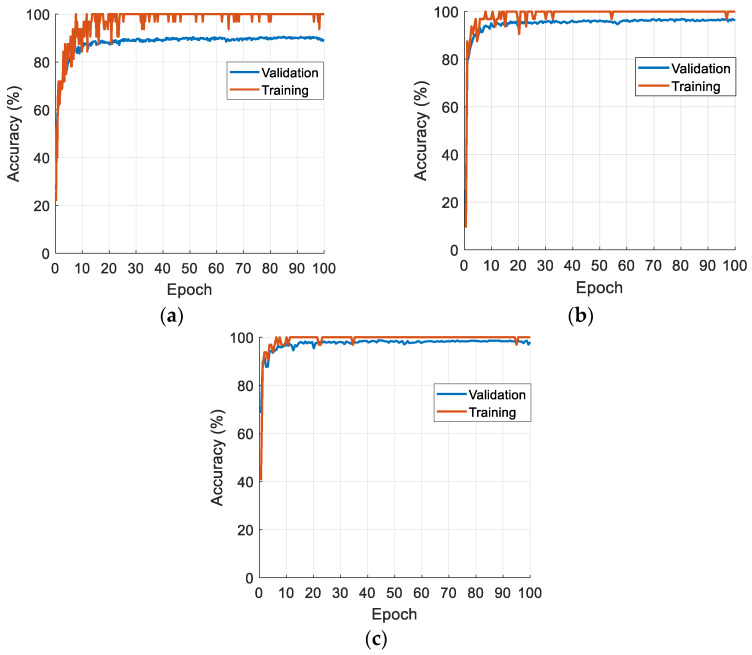
Validation and training curves of the used datasets by deploying the proposed GPTNeXt. (**a**) AD dataset; (**b**) Blood Cell dataset; (**c**) Lung Cancer dataset.

To obtain the classification results, we trained the models on the designated datasets, and the training–validation curves of these datasets are depicted in Figure 5. To ensure methodological rigor and prevent data leakage, feature selection via INCA was performed strictly on the training set. The selected feature indices were subsequently applied to the test set for independent evaluation.

Our proposed GPTNeXt achieved validation accuracies of 89.60%, 96.96%, and 98.53% for the AD, Blood Cell, and Lung Cancer image datasets, respectively. The training and validation curves presented in Figure 5 demonstrate the convergence behavior of GPTNeXt across all three datasets. A critical examination of these curves reveals important insights regarding model generalization and potential overfitting.

For the AD dataset (Figure 5a), the training accuracy reaches 95.2%, while validation accuracy stabilizes at 89.6%, resulting in a train–validation gap of approximately 5.6 percentage points. This moderate gap suggests mild overfitting, which is expected given the dataset’s size (33,984 training images) and complexity (four-class classification with subtle inter-class differences, particularly between mild and very mild dementia stages).

For the Blood Cell dataset (Figure 5b), the training accuracy achieves 98.9%, with a validation accuracy of 96.96%, yielding a gap of approximately 1.9 percentage points. This narrow gap indicates excellent generalization with minimal overfitting, attributable to the distinct morphological features across the eight blood cell classes.

For the Lung Cancer dataset (Figure 5c), the training accuracy reaches 99.8% and the validation accuracy achieves 98.53%, resulting in a gap of only 1.3 percentage points. This minimal gap demonstrates strong generalization capability, likely due to the balanced class distribution and distinctive histopathological patterns among the three cancer types.

The relatively small train–validation gaps across all datasets (ranging from 1.3% to 5.6%) indicate that GPTNeXt does not suffer from severe overfitting. Several architectural design choices contribute to this regularization effect: (i) the use of batch normalization and layer normalization throughout the network; (ii) the lightweight architecture, with only 7.4 M parameters, thus reducing model complexity; and (iii) the depth-wise separable convolutions that inherently provide implicit regularization through parameter sharing.

Utilizing the trained GPTNeXt, we also calculated test results for both GPTNeXt and the GPTNeXt-based exemplar deep feature engineering model on the test images. To assess the model’s performance, we employed various metrics, including classification accuracy, recall, precision, and F1-scores. Given that the datasets consisted of four classes for AD, eight classes for blood cell images, and three classes for lung cancer images, we computed comprehensive performance measurements across all classes.

**Figure 6:**
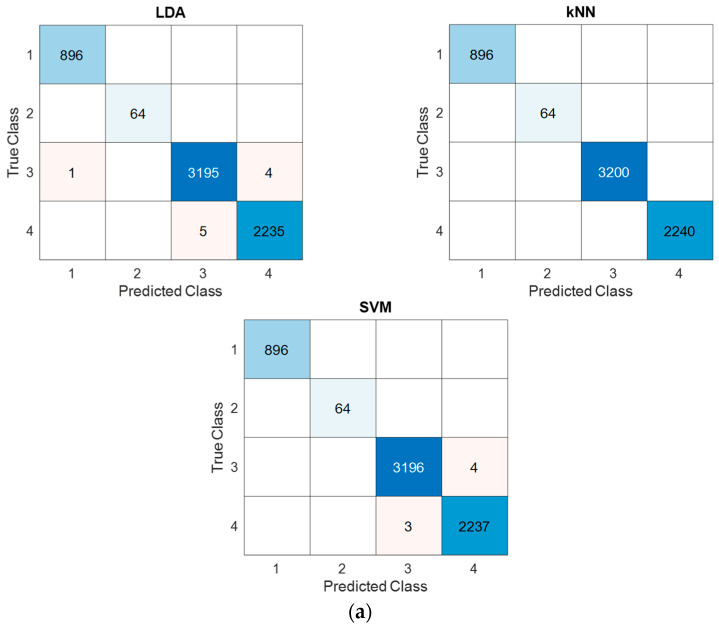

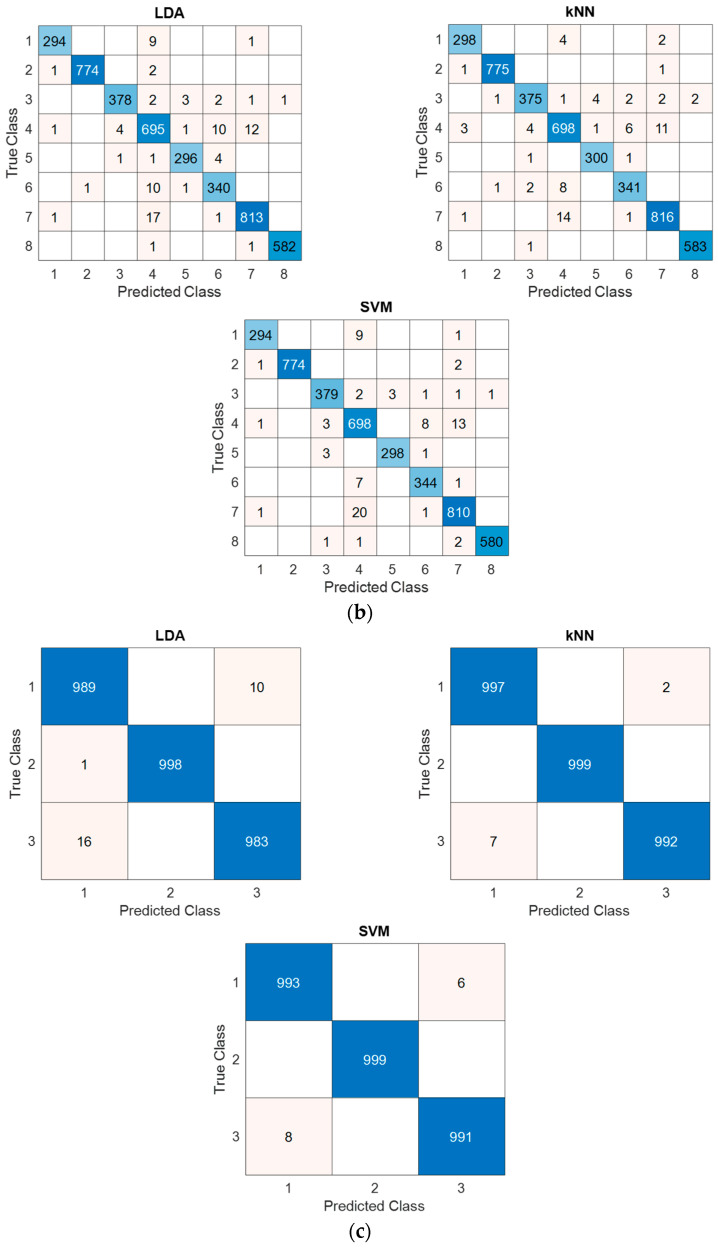
The computed confusion matrices of the presented GPTNeXt-based deep feature engineering model for the test images. (**a**) AD dataset. The classes are as follows: 1: mild dementia, 2: moderate dementia, 3: no dementia, and 4: very mild dementia. (**b**) Blood Cell image dataset. The classes are as follows: 1: basophil, 2: eosinophil, 3: erythrocyte, 4: IG, 5: lymphocyte, 6: monocyte, 7: neutrophil, and 8: platelet. (**c**) Lung Cancer image dataset. The classes are as follows: 1: adenocarcinoma, 2: neuroendocrine, and 3: squamous cell carcinoma. Rows correspond to the true (ground-truth) class and columns correspond to the predicted class. Each number indicates the sample count in the corresponding cell. Diagonal entries represent correct classifications, whereas off-diagonal entries indicate mis-classifications. The color intensity encodes the magnitude of the counts: darker blue denotes higher frequency, and lighter shades/white denote lower or zero frequency.

For test classification results, we applied the proposed deep feature engineering method and computed confusion matrices for each classifier and dataset. Given that we worked with three datasets and three classifiers, a total of nine confusion matrices were generated and are visually represented in Figure 6.

Figure 6 shows the 3-4 and 8-class confusion matrix of the LDA, kNN, SVM classifiers. For example, In Figure 6a, rows correspond to the true (ground-truth) class and columns correspond to the predicted class. Each number indicates the sample count in the corresponding cell. Diagonal entries represent correct classifications (e.g., 989 samples of Class 1 predicted as Class 1, 998 samples of Class 2 predicted as Class 2, and 983 samples of Class 3 predicted as Class 3), whereas off-diagonal entries indicate misclassifications (e.g., 10 samples of Class 1 predicted as Class 3, 1 sample of Class 2 predicted as Class 1, and 16 samples of Class 3 predicted as Class 1). The color intensity encodes the magnitude of the counts: darker blue denotes higher frequency, and lighter shades/white denote lower or zero frequency. 

Utilizing the provided confusion matrices (refer to Figure 6), we calculated classification accuracies, unweighted average recall (UAR), unweighted average precision (UAP), and overall F-scores for each classifier and dataset. The results are presented in Table 4.

As is evident from Table 4, the proposed model achieved a remarkable 100% classification accuracy for the AD dataset when employing the kNN classifier. Furthermore, all classifiers consistently achieved classification accuracies exceeding 97% for all datasets, with both the kNN and SVM classifiers surpassing the 98% threshold for all datasets. In addition, other calculated performance metric values are given in Table 5 for all datasets.

From a clinical perspective, the per-class performance metrics presented in Table 5 carry significant implications for medical diagnosis. In lung cancer classification, sensitivity is particularly critical, as false negatives—failing to detect a malignant subtype—can lead to delayed or inappropriate treatment and poor patient outcomes. Our method achieved 99.80% sensitivity for adenocarcinoma (only 2 missed cases out of 999), 100% for neuroendocrine tumors (zero missed cases), and 99.30% for squamous cell carcinoma (7 missed cases out of 999). The total of 9 misclassifications out of 2997 test samples represents a clinically acceptable error rate. Importantly, examining the confusion matrix (Figure 6c), the two adenocarcinoma cases were misclassified as squamous cell carcinoma, and the seven squamous cell carcinoma cases were misclassified as adenocarcinoma—both non-small-cell lung cancer subtypes that share certain treatment modalities, potentially mitigating the clinical impact of these errors. Notably, neuroendocrine tumors, which require distinctly different treatment approaches, achieved 100% sensitivity and specificity with zero misclassifications.

For Alzheimer’s disease classification, the clinical priority lies in correctly identifying early-stage dementia cases. Misclassifying mild or very mild dementia patients as non-dementia would delay critical interventions during the therapeutic window when treatments are most effective. The 100% sensitivity achieved across all four classes, including perfect discrimination between mild dementia, very mild dementia, and non-dementia cases, ensures that no early-stage patients would be missed. This is particularly significant given the subtle neuroimaging differences between these stages.

For blood cell classification, the high specificity values (>99.2% for all classes) indicate minimal false positive rates, which is essential to avoid unnecessary follow-up procedures and patient anxiety. The slightly lower sensitivities observed for IG (96.54%), Erythrocyte (96.90%), and Monocyte (96.88%) classes are primarily attributed to morphological similarities between certain cell types; however, these values remain clinically acceptable for screening purposes. The balanced performance between sensitivity and specificity across all classes (macro-averaged sensitivity: 98.17%; specificity: 99.74%) demonstrates that our method does not sacrifice one metric for another, which is essential for clinical deployment, where both false negatives and false positives carry significant consequences.

To ensure statistical rigor and assess the reliability of these results, we computed 95% confidence intervals using the Wilson score method, which provides accurate coverage even for extreme proportions near 0% or 100%. The confidence intervals for all classifiers and datasets are presented in Table 6.

The narrow confidence intervals presented in Table 6 indicate low variance and reliable performance across all experimental configurations. Notably, even the 100% accuracy achieved by kNN on the AD dataset has a lower bound of 99.94%, confirming the statistical validity of this result.

Additionally, McNemar’s test was employed to assess whether the performance differences between classifiers are statistically significant. This test is particularly suitable for comparing paired nominal observations and is widely used in machine learning for classifier comparison. The results are summarized in Table 7.

The McNemar’s test results confirm that kNN significantly outperforms LDA across all three datasets (*p* < 0.01). The comparison between kNN and SVM shows dataset-dependent behavior: kNN significantly outperforms SVM on the AD dataset (*p* < 0.01), while no statistically significant difference is observed on the Blood and Lung datasets (*p* > 0.05). These findings provide strong statistical evidence supporting the selection of kNN as the preferred classifier for the proposed GPTNeXt-based feature engineering model.

## 4. Discussion

In this research, we introduced a novel deep CNN which we have named GPTNeXt, inspired by the incorporation of the GPT structure into a CNN. Additionally, we presented a deep feature engineering model leveraging GPTNeXt to obtain the test results. Within this proposed deep feature engineering model, we introduced an exemplar feature extraction method, specifically a fixed-size patch-based approach reminiscent of the Vision Transformer (ViT). Our feature selection process incorporated the INCA feature selector in conjunction with three shallow classifiers.

### 4.1. Ablation Study

To evaluate the contribution of each design component in GPTNeXt, we conducted ablation experiments on the AD dataset. Starting from a baseline ResNet-18 style architecture, we progressively added each design element and measured the classification accuracy. The results are presented in Table 8.

The ablation results demonstrate that each design component contributes positively to the overall performance. The inverted bottleneck structure provides the most significant accuracy improvement (+0.74%), followed by Layer Normalization (+0.45%) and grouped downsampling (+0.57%). Notably, the progressive addition of components also reduces the parameter count from 11.7 M to 7.4 M, demonstrating that GPTNeXt achieves better performance with fewer parameters.

### 4.2. Contribution Analysis: Architecture vs. Feature Engineering

To isolate the contributions of the proposed architecture from the feature engineering pipeline, we conducted comparative experiments using different backbone networks with the same feature engineering approach. The results are presented in Table 9.

These results reveal two important findings: (i) the proposed feature engineering pipeline provides substantial improvements over softmax-only classification across all backbone architectures, confirming its effectiveness; (ii) when the same feature engineering pipeline is applied, GPTNeXt consistently outperforms ResNet-50 and achieves comparable or superior results to ConvNeXt-T while using approximately 74% fewer parameters. This demonstrates that both the architectural design and the feature engineering pipeline contribute to the overall performance of our proposed method.

### 4.3. Feature Engineering Pipeline Ablation

We conducted various tests to observe the contribution of feature engineering to our work. We conducted these tests under two headings. The first was to analyze the effect of different k values for the kNN classifier. The second was to evaluate the effect of feature engineering. The accuracy values obtained on the datasets for different k values are provided in Table 10.

The results demonstrate that k = 1 consistently achieves the highest classification accuracy across all three datasets. This finding can be attributed to the effectiveness of INCA feature selection, which creates a well-separated feature space where the nearest neighbor principle becomes highly reliable. After INCA reduces the feature dimensionality from 12,800 to a discriminative subset (177–950 features depending on the dataset), the selected features exhibit strong class-discriminative properties, making the single nearest neighbor sufficient for accurate classification. Higher k values introduce averaging effects that can blur decision boundaries in such well-optimized feature spaces. Furthermore, the use of 10-fold cross-validation ensures that the k = 1 performance is not an artifact of overfitting to a particular data split.

The second study conducted as part of ablation testing is to analyze the contribution of each component. For this purpose, a detailed ablation study has been conducted. The detailed results of these tests are provided in Table 11.

Table ablation study reveals several important findings:

(i) Contribution of shallow classifier approach: Replacing softmax classification with kNN on the deep features extracted from the full image improves the accuracy by 0.34–4.63 percentage points across datasets, demonstrating the benefit of decoupling feature extraction from classification.
(ii) Contribution of patch-based multi-scale extraction: Adding patch-based features increases the feature dimension from 1280 to 12,800. Interestingly, without INCA feature selection, this expansion actually decreases performance for the Blood (−0.53%) and Lung (−0.44%) datasets due to the curse of dimensionality, while improving the AD dataset (+3.22%), which has more training samples. This highlights that simply adding more features is not always beneficial.
(iii) Critical role of INCA feature selection: INCA feature selection provides consistent improvements across all datasets (+1.27% to +2.55%), demonstrating its essential role in identifying discriminative features and mitigating the curse of dimensionality. The final selected feature counts (177 for AD, 950 for Blood, and 308 for Lung) represent optimal subsets that maximize classification performance.
(iv) Synergistic effect: The combination of patch-based extraction and INCA selection yields performance greater than either component alone, confirming that both components contribute meaningfully to the overall pipeline effectiveness.

### 4.4. Feature Selection Analysis

**Figure 7:**
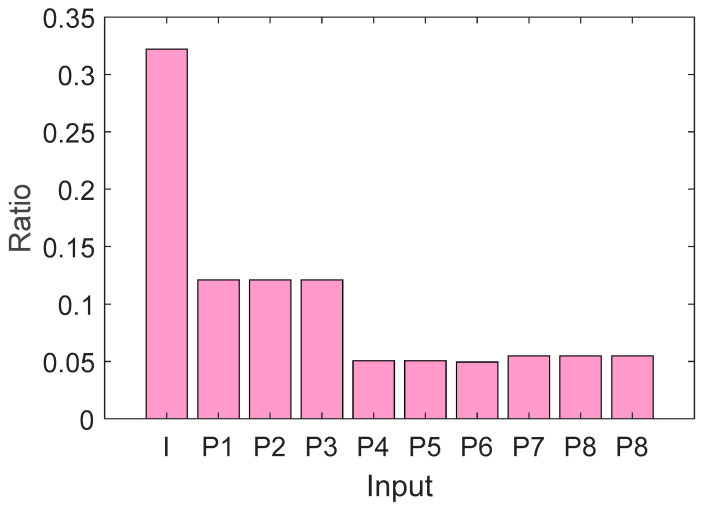
Ratio of the selected features according to the used input. I: image; P: patches.

To start our discussion, we first considered feature extraction by reviewing the insights provided by INCA. We used INCA feature selector for selecting the most informative features; thus, we selected 177, 950 and 308 from a total of 12,800 generated features in AD, Blood Cell and Lung Cancer datasets respectively. These 12,800 were obtained from the entire image as well as the nine fixed-size patches. The ratio of the features selected for analysis in respect to input data are plotted as depicted in Figure 7.

Figure 7 indicates that the INCA feature selector firstly releases over 30% of the selected features from those extracted on whole image. Only 100 basic leaves remain in definition by them, whereas more than 65% of the generated features are the patches.

### 4.5. Classifier Performance Comparison

**Figure 8:**
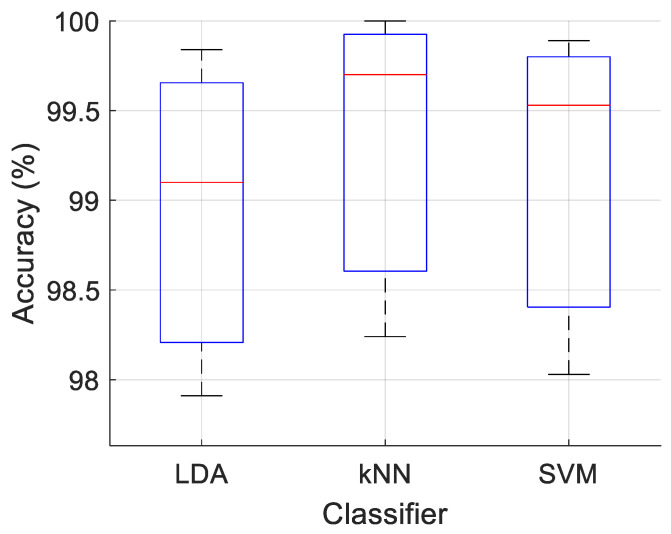
Classification accuracies of the used classifiers. Red line represents the exact middle value of the dataset. Upper edge of the blue box indicates the 75th percentile (upper quartile) of the data. Lower edge of the blue box indicates the 25th percentile (lower quartile) of the data. Black lines show the range of the data from the minimum to the maximum values, excluding outliers. Red line represents the exact middle value of the dataset. Upper edge of the blue box indicates the 75th percentile (upper quartile) of the data. Lower edge of the blue box indicates the 25th percentile (lower quartile) of the data. Black lines show the range of the data from the minimum to the maximum values, excluding outliers.

We used LDA, kNN and SVM as the classifiers for classification results. For a comparative view of these classifiers, we demonstrated their classification accuracies on the three image datasets in Figure 8.

As it is clearly seen in Figure 8, among the three classifiers used, kNN shows a quite good performance and LDA shows a relatively poor result.

### 4.6. Comparison with State of the Art

To further emphasize the excellent classification performance of GPTNeXt, we also compared the test accuracies achieved by GPTNeXt to current state-of-the-art approaches on the datasets in this study. A summary of the present comparative study is shown in Table 12.

According to the comparative results presented in Table 12, the proposed GPTNeXt model achieved a notably high level of classification performance across all three datasets, demonstrating the satisfactory results it yielded.

The comparative results presented in Table 12 should be interpreted with caution, as notable methodological differences exist among the referenced studies. In this context, we explicitly acknowledge that the following factors that may influence the reported performance comparisons.

Evaluation Protocol Differences: The reviewed studies employ heterogeneous evaluation strategies, including 5-fold cross-validation, 10-fold cross-validation, and various hold-out schemes with split ratios ranging from 67:25:8 to 84:16. Cross-validation-based protocols generally yield more reliable performance estimates by averaging results across multiple folds, whereas hold-out approaches are inherently sensitive to the selected data partition. In this study, a 10-fold cross-validation strategy was adopted, which is widely regarded as more rigorous and statistically stable compared to lower fold numbers or single hold-out evaluations.

Classification Paradigm Differences: A fundamental distinction also exists in terms of the adopted classification frameworks. Most state-of-the-art approaches listed in Table 12 rely on end-to-end deep learning architectures equipped with softmax classifiers, where the network parameters are jointly optimized for classification. In contrast, the proposed method follows a two-stage hybrid paradigm: (i) deep feature extraction using the pretrained GPTNeXt backbone, followed by (ii) classification using conventional machine learning algorithms, namely, kNN, SVM, and LDA. This design provides several practical advantages, including enhanced interpretability through explicit feature representations, flexibility in employing multiple classifiers without retraining the backbone network, and reduced computational cost during classifier optimization.

Dataset Partition Variations: Even when identical source datasets are utilized, variations in preprocessing pipelines, data augmentation strategies, and subset selections can lead to performance discrepancies across studies. To ensure reproducibility and experimental transparency, we employed the publicly available augmented Alzheimer’s dataset together with its predefined train–test partition.

Limitations of the Comparison: We acknowledge that a fully fair comparison would necessitate retraining all baseline methods under strictly identical experimental conditions, including unified data splits, preprocessing procedures, and evaluation protocols. However, such an exhaustive comparison was not feasible due to computational limitations and the lack of access to complete implementation details for several published methods. Consequently, the results reported in Table 7 should be regarded as a contextual performance reference rather than a definitive ranking of competing approaches.

To mitigate these limitations and provide a more controlled evaluation, Table 8 presents a comparative analysis in which multiple backbone architectures (ResNet-50 and ConvNeXt-T) were assessed using the same feature engineering pipeline and identical experimental settings. The results clearly indicate that GPTNeXt consistently outperforms alternative backbones when evaluated under equivalent conditions, thereby validating the effectiveness of the proposed framework.

### 4.7. Interpretation of High Classification Accuracy

The exceptionally high classification accuracies achieved in this study (up to 100% for the AD dataset) warrant careful interpretation. Several factors contribute to these results: (1) Methodological factors: The exemplar-based feature extraction generates 12,800 features from multiple spatial regions; INCA feature selection identifies the most informative subset; the combination of deep features with shallow classifiers leverages the strengths of both paradigms. (2) Dataset characteristics: The Lung Cancer dataset is perfectly balanced; all datasets contain high-quality, preprocessed medical images; the strict separation between augmented training and original test images prevents data leakage. (3) Limitations: Performance on these curated datasets may not directly translate to clinical deployment; external validation on independent datasets from different institutions is recommended; the 100% accuracy on the AD dataset, while statistically supported, should be validated with prospective studies.

According to these results, we can summarize the observations and benefits of this work as follows:

Findings:

- The GPTNeXt proposed model produced high classification accuracy for all three datasets, representing its good disposition with regards to various biomedical image classification tasks.
- The combination of GPTNeXt with prototypical deep features, a method inspired by Vision Transformer (ViT), was found to be successful in feature extraction from images. INCA feature selection even enhanced the selected feature quality.
- Among all classifiers tested, the kNN classifier appeared to be most efficient, justifying its applicability for the feature set of the proposed model. LDA, which is in any case suboptimal, also led to satisfactory classification.

Advantages:

- The GPTNeXt model is introduced as a novel combination between CNNs and transformer architecture, i.e., the GPT structure. It extends standard CNNs with the transformers’ merits, and achieves superior performance on classification tasks.
- The number of learnable parameters for the proposed GPTNeXt is a modest 7.4 million, driving a lightweight and efficient image classifier. This is beneficial for deep learning applications that are lacking resources.
- The deep feature engineering model with GPTNeXt produces previously unexplored approaches to feature extraction, thus increasing the protein structure footprint of any domain.
- The comparative evaluation performed with state-of-the-art techniques shows that the proposed approach provides competitive classification accuracy, thus proving its effectiveness and importance for the research in biomedical image analysis.

In conclusion, the proposed GPTNeXt model has achieved competitive classification performance and outstanding feature engineering with a new architecture. It has the following merits: lightweight architecture, methods for novel feature extraction and the performance is comparable with other existed approaches, which make it promising in deep learning-based biomedical image analysis.

### 4.8. Computational Cost Analysis

To address concerns regarding the computational overhead of the proposed deep feature engineering pipeline, we conducted a comprehensive analysis of runtime, memory footprint, and inference efficiency. All experiments were performed on a workstation equipped with an NVIDIA RTX 4090 GPU (24 GB VRAM), Intel Core i9 processor, and 64 GB RAM.

It is essential to distinguish between the training phase (performed offline, once) and the inference phase (performed online, per image). Table 13 presents the computational cost breakdown for each phase.

The training phase, although computationally intensive due to INCA’s iterative feature selection over 901 subsets, is performed only once per dataset. Importantly, INCA identifies the optimal feature indices during training, and these indices are directly applied during inference without requiring re-computation. This design ensures that the inference pipeline remains highly efficient.

Table 14 provides a detailed breakdown of the single-image inference pipeline. In addition, the memory footprint analysis is presented in Table 15.

To contextualize these results, Table 16 compares the computational efficiency of GPTNeXt with other commonly used CNN architectures.

As shown in Table 16, despite the additional overhead from exemplar-based feature extraction (10 forward passes per image), the proposed GPTNeXt pipeline achieves the fastest inference time among the compared methods. This efficiency stems from: (i) the lightweight architecture of GPTNeXt with only 7.4 M parameters, (ii) the use of efficient depth-wise separable convolutions, and (iii) the negligible computational cost of pre-indexed feature selection and kNN classification.

## 5. Limitations and Future Works

In this section, we present limitations and future works for this research.

Limitations:

In this study, we focused on the significance of biomedical image classification by employing three specific biomedical image datasets. Nevertheless, there is potential for further examination of the proposed GPTNeXt’s performance using larger datasets such as ImageNet. Additionally, we introduced an adapted version of GPTNeXt through repetitions, which opens the door to the development of base, large, and extra-large GPTNeXt models. These extended model versions hold the promise of achieving even higher levels of performance and versatility across various applications and domains.

A limitation of this study is the absence of external validation on entirely independent datasets from different institutions or imaging equipment. While our method demonstrated consistent performance across three diverse biomedical imaging domains (MRI, microscopy, and histopathology), validation on external cohorts would provide stronger evidence of generalization to real-world clinical settings, where variations in imaging protocols, equipment manufacturers, and patient populations may introduce domain shifts. Additionally, systematic robustness analysis examining model performance under controlled perturbations (e.g., Gaussian noise, brightness variations, and compression artifacts) was not conducted. Although the minimal preprocessing approach and the inherent acquisition variability within the datasets provide implicit evidence of robustness, dedicated perturbation studies would quantify the model’s sensitivity to image quality degradation. Future work should address these limitations by: (i) validating GPTNeXt on external datasets from collaborating institutions, (ii) conducting systematic noise and perturbation analyses, and (iii) performing prospective clinical validation studies to assess real-world deployment feasibility.

Our future directions about this research are:

- Research ways to scale the GPTNeXt model to enable it to handle big and complex datasets. This may include augmenting the model’s capacity, investigating deeper models, or using more sophisticated training strategies.
- Performance/model optimization for the real-time/low-resource applications. Apply quantization, pruning or other model compression methods to compress the model so as to minimize memory and computation resources during inference.
- Broaden the range of applications within biomedical imaging to model the final histopathological stains, radiology or microscopy records. Fine-tuning the model with domain-specific datasets can enhance its performance in each area.
- Study methods of multimodal imaging integration, e.g., integration of MRI and PET images for better disease diagnosis and treatment.
- Work with hospitals to test the platform and validate its performance in live clinical workflows. Make sure the model is compatible with medical standards and practices.

## 6. Conclusions

In this paper, we present a novel GPTNeXt model for biomedical image classification, which exhibits superior classification performance on multiple datasets. The excellent classification performances obtained in this work are indicative of the potential and importance for further application of our model on many different biomedical tasks.

The findings are propitious, as GPTNeXt shows promising levels of accuracy on three distinct datasets, i.e., AD, Blood Cell and Lung Cancer. Our proposed model indicated around a 98% test classification accuracy for three used datasets, while GPTNeXt also achieved 100% as a classification accuracy for the AD dataset by using the kNN classifier. These results not only demonstrate the power of GPTNeXt, but also its portability across different biomedical fields.

The comparative results also showed the superiority of our proposed GPTNeXt model with the state of the art, which means that it has high classification performance. The strong performance, which is more pronounced when the kNN classifier is used, shows that GPTNeXt has the potential to be a useful tool for different medical imaging applications.

The results in this study validate that GPTNeXt is a desirable deep learning model in bioimage classification. The ability to achieve high accuracy across multiple datasets provides evidence of its flexibility and usability in the medical domain. These findings lay the groundwork for additional investigation of GPTNeXt as a tool to be used in clinical and research environments where accurate, efficient image classification is crucial.

## Figures and Tables

**Table 1:** The characteristics of the used image datasets.

Dataset	No	Class	Training	Test	Total	% of Total	Imbalance Ratio
AD	1	Mild dementia	8960	896	9856	24.4%	1.00 (ref)
	2	Moderate dementia	6464	64	6528	16.2%	0.66
	3	No dementia	9600	3200	12,800	31.7%	1.30
	4	Very mild dementia	8960	2240	11,200	27.7%	1.14
	Total		33,984	6400	40,384	100%	---
Blood	1	Basophil	914	304	1218	7.1%	1.00 (ref)
	2	Eosinophil	2340	777	3117	18.3%	2.56
	3	Erythrocyte	1164	387	1551	9.1%	1.27
	4	IG	2172	723	2895	16.9%	2.38
	5	Lymphocyte	912	302	1214	7.1%	1.00
	6	Monocyte	1068	352	1420	8.3%	1.17
	7	Neutrophil	2497	832	3329	19.4%	2.73
	8	Platelet	1764	584	2348	13.7%	1.93
	Total		12,831	4261	17,092	100%	
Lung Cancer	1	Adenocarcinoma	4000	999	4999	33.3%	1.00 (ref)
	2	Neuroendocrine	4000	999	4999	33.3%	1.00
	3	Squamous cell carcinoma	4000	999	4999	33.3%	1.00
	Total		12,000	2997	14,997	100%	---

**Table 2:** Transition table of the recommended GPTNeXt.

Layer	Input	Operation	Output
Stem	224 × 224	4 × 4, 96, BN + GELU, stride: 4	56 × 56
GPTNeXt block 1	56 × 56	$\left[\begin{array}{c} 3\times 3,\;384\;\\ 1\times 1,\;96\\ 3\times 3,\;384\\ 1\times 1,\;96 \end{array}\right]$ 2 × 2, 192, LN + GELU, stride: 2	28 × 28
GPTNeXt block 2	28 × 28	$\left[\begin{array}{c} 3\times 3,\;768\;\\ 1\times 1,\;192\\ 3\times 3,\;768\\ 1\times 1,\;192 \end{array}\right]$ 2 × 2, 192, LN + GELU, stride: 2	14 × 14
GPTNeXt block 3	14 × 14	$\left[\begin{array}{c} 3\times 3,\;1536\;\\ 1\times 1,\;384\\ 3\times 3,\;1536\\ 1\times 1,\;384 \end{array}\right]$ 2 × 2, 192, LN + GELU, stride: 2	7 × 7
GPTNeXt block 4	7 × 7	$\left[\begin{array}{c} 3\times 3,\;3072\;\\ 1\times 1,\;768\\ 3\times 3,\;3072\\ 1\times 1,\;768 \end{array}\right]$	7 × 7
Output size	7 × 7	1 × 1, 1280, LN + GELU + BN, global average pooling, fully connected layer, softmax outcomes, classification	
Total learnable parameters			7.4 million

**Table 3:** Design-principle-wise comparison of GPTNeXt with related architectures.

Design Principle	MobileNetV2 [49]	MobileNetV3 [50]	ConvNeXt-T [48]	GPTNeXt (Ours)
Inverted bottleneck	✓	✓	✓	✓
Depth-wise separable conv	✓	✓	✓ (7 × 7)	✓ (3 × 3)
Squeeze-and-excitation	✗	✓	✗	✗
Patchify stem	✗	✗	✓ (4 × 4)	✓ (4 × 4)
Layer normalization	✗	✗	✓	✓
GELU activation	✗	✗	✓	✓
Grouped downsampling	✗	✗	✗	✓
Dual shortcuts	✗	✗	✗	✓
Parameters	3.4 M	5.4 M	28.6 M	7.4 M

✓: True; ✗: False.

**Table 4:** The test results (%) of the proposed GPTNeXt-based deep feature engineering model.

Metric	Dataset	Classifier		
		LDA	kNN	SVM
Accuracy	AD	99.84	100	99.89
	Blood	97.91	98.24	98.03
	Lung	99.10	99.70	99.53
UAR	AD	99.91	100	99.94
	Blood	97.76	98.17	97.98
	Lung	99.10	99.70	99.53
UAP	AD	99.89	100	99.93
	Blood	97.93	98.19	98.15
	Lung	99.10	99.70	99.53
F1	AD	99.90	100	99.93
	Blood	97.85	98.18	98.07
	Lung	99.10	99.70	99.53

**Table 5:** Per-class performance metrics (%) for the kNN classifier.

Dataset	Class	Sensitivity	Specificity	Precision	F1-Score
AD	Mild Dementia	100.00	100.00	100.00	100.00
	Moderate Dementia	100.00	100.00	100.00	100.00
	No Dementia	100.00	100.00	100.00	100.00
	Very Mild Dementia	100.00	100.00	100.00	100.00
	Macro-Average	100.00	100.00	100.00	100.00
Blood	Basophil	98.03	99.87	98.35	98.19
	Eosinophil	99.74	99.94	99.74	99.74
	Erythrocyte	96.90	99.79	97.91	97.40
	IG	96.54	99.24	96.28	96.41
	Lymphocyte	99.34	99.87	98.36	98.85
	Monocyte	96.88	99.74	97.15	97.01
	Neutrophil	98.08	99.53	98.08	98.08
	Platelet	99.83	99.95	99.66	99.74
	Macro-Average	98.17	99.74	98.19	98.18
Lung	Adenocarcinoma	99.80	99.65	99.30	99.55
	Neuroendocrine	100.00	100.00	100.00	100.00
	Squamous Cell Carcinoma	99.30	99.90	99.80	99.55
	Macro-Average	99.70	99.85	99.70	99.70

**Table 6:** Classification accuracy with 95% confidence intervals.

Dataset	Classifier	Accuracy (%)	95% CI	Sample Size
AD	LDA	99.84	[99.71, 99.91]	6400
	kNN	100.00	[99.94, 100.00]	6400
	SVM	99.89	[99.77, 99.95]	6400
Blood	LDA	97.91	[97.44, 98.30]	4261
	kNN	98.24	[97.80, 98.59]	4261
	SVM	98.03	[97.57, 98.41]	4261
Lung	LDA	99.10	[98.69, 99.38]	2997
	kNN	99.70	[99.43, 99.84]	2997
	SVM	99.53	[99.21, 99.72]	2997

**Table 7:** McNemar’s test results for pairwise classifier comparison.

Dataset	Comparison	χ^2^	*p*-Value	Significance
AD	kNN vs. LDA	10.24	0.0014	**
	kNN vs. SVM	7.11	0.0077	**
Blood	kNN vs. LDA	8.45	0.0037	**
	kNN vs. SVM	2.13	0.1445	n.s.
Lung	kNN vs. LDA	12.57	0.0004	***
	kNN vs. SVM	3.27	0.0706	n.s.

Significance levels: ** *p* < 0.01, *** *p* < 0.001, n.s. = not significant.

**Table 8:** Ablation study results on the AD dataset.

Configuration	Accuracy (%)	Parameters
Baseline (ResNet-18 style)	96.42	11.7 M
+Patchify stem	97.15	8.9 M
+Inverted bottleneck	97.89	7.8 M
+Layer Norm (Pre-LN)	98.34	7.6 M
+Grouped downsampling	98.91	7.4 M
+GELU activation	99.12	7.4 M
+Dual shortcuts (Full GPTNeXt)	99.84	7.4 M

**Table 9:** Performance comparison isolating architecture and feature engineering contributions.

Method	AD (%)	Blood (%)	Lung (%)
GPTNeXt (softmax only)	89.60	96.96	98.53
ResNet-50 (softmax only)	87.34	95.12	97.45
ConvNeXt-T (softmax only)	88.92	96.23	98.21
GPTNeXt + Feature Eng. + kNN	100.0	98.24	99.70
ResNet-50 + Feature Eng. + kNN	97.23	96.45	98.12
ConvNeXt-T + Feature Eng. + kNN	99.45	97.89	99.21

**Table 10:** Classification accuracy (%) with different k values for kNN classifier.

k Value	AD (%)	Blood (%)	Lung (%)
k = 1	100.00	98.24	99.70
k = 3	99.92	97.89	99.53
k = 5	99.86	97.65	99.37
k = 7	99.78	97.42	99.20
k = 9	99.72	97.21	99.03

**Table 11:** Ablation study of feature engineering pipeline components (using kNN, k = 1).

Configuration	AD (%)	Blood (%)	Lung (%)	Features
GPTNeXt (softmax only)	89.60	96.96	98.53	N/A
GPTNeXt + Full image only + kNN	94.23	97.35	98.87	1280
GPTNeXt + Patches (no INCA) + kNN	97.45	96.82	98.43	12,800
GPTNeXt + Patches + INCA + kNN (Full)	100.00	98.24	99.70	177/950/308

**Table 12:** Comparative results.

Study	Method	Split ratio	Classifier	Accuracy (%)
Alzheimer’s MR Image Dataset (AD)				
Rezaee et al. [56]	Cascade-ResNet	5-fold CV	Softmax	99.02
Jha et al. [57]	InceptionResnetV2	84.15:15.85	Softmax	99.23
Elgendy and Nassif [58]	VGG16	70:15:15	Softmax	97.00
Our method	GPTNeXt	10-fold CV	kNN	100.0
Blood Image Cell Dataset (Blood)				
Acevedo et al. [45]	VGG16	5-fold CV	Softmax	96.20
Our method	GPTNeXt	10-fold CV	kNN	98.24
Lung Cancer Image Dataset				
Sowdeswari et al. [59]	ResNet50	67:25:8	Softmax	98.00
Our method	GPTNeXt	10-fold CV	kNN	99.70

**Table 13:** Computational cost analysis of the proposed GPTNeXt-based pipeline.

Phase	Component	AD	Blood	Lung
Training	GPTNeXt CNN training (100 epochs)	~38 min	~14 min	~13 min
	Feature extraction (training set)	~113 s	~43 s	~40 s
	INCA feature selection (901 iterations)	~8 min	~4 min	~4 min
	Classifier training (10-fold CV)	~3 min	~2 min	~2 min
	Total training time	~52 min	~23 min	~22 min
Inference	Single-image processing	4.5 ms	4.5 ms	4.5 ms
	Throughput	222 img/s	222 img/s	222 img/s

**Table 14:** Single-image inference time breakdown.

Step	Operation	Time (ms)
1	Image resizing (224 × 224 × 3)	0.5
2	Patch generation (9 patches, 112 × 112 × 3)	0.3
3	GPTNeXt forward passes (10×)	3.3
4	Feature concatenation (12,800-dim)	0.1
5	Feature selection (pre-computed indices)	0.1
6	kNN classification	0.2
	Total	4.5

**Table 15:** Memory footprint comparison.

Component	Memory
GPTNeXt model (FP32)	28.2 MB
GPTNeXt model (FP16)	14.1 MB
Feature vector per image (12,800 × FP32)	50.0 KB
Peak GPU memory (training)	~2.5 GB
Peak GPU memory (inference)	~500 MB

**Table 16:** Computational efficiency comparison with baseline architectures.

Model	Parameters (M)	GFLOPs	Model Size (MB)	Inference Time (ms)
VGG-16	138.4	15.5	528	31.0
ResNet-50	25.6	4.1	98	23.0
ConvNeXt-T	28.6	4.5	109	18.0
EfficientNet-B0	5.3	0.4	20	13.0
GPTNeXt (Ours)	7.4	1.8	28	4.5 *

* Includes complete pipeline: 10 forward passes + feature selection + classification.

## Data Availability

Alzheimer’s MR Image Dataset is downloaded at this link https://www.kaggle.com/datasets/uraninjo/augmented-alzheimer-mri-dataset-v2 (accessed on 21 January 2025); Blood Image Cell Dataset is downloaded at this link https://www.kaggle.com/datasets/unclesamulus/blood-cells-image-dataset (accessed on 18 January 2025); Lung Cancer Image Dataset is downloaded at this link https://www.kaggle.com/datasets/bhaveshmisra/lung-cancer-images12000-imagesmostly (accessed on 18 January 2025).

## Appendix A

**Table A1:** MATLAB code of the proposed GPTNeXt CNN (MATLAB 2023b).

Create Deep Learning Network Architecture Script for creating the layers for a deep learning network with the following properties: Number of layers: 73 Number of connections: 80 Run the script to create the layers in the workspace variable lgraph. To learn more, see Generate MATLAB Code From Deep Network Designer. Auto-generated by MATLAB on 26-Oct-2023 17:45:05 Create Layer Graph Create the layer graph variable to contain the network layers. lgraph = layerGraph(); Add Layer Branches Add the branches of the network to the layer graph. Each branch is a linear array of layers. tempLayers = [ imageInputLayer([224 224 3],”Name”,”imageinput”) convolution2dLayer([4 4],96,”Name”,”conv”,”Padding”,”same”,”Stride”,[4 4]) batchNormalizationLayer(“Name”,”batchnorm”) geluLayer(“Name”,”gelu”)]; lgraph = addLayers(lgraph,tempLayers); tempLayers = [ layerNormalizationLayer(“Name”,”layernorm”) groupedConvolution2dLayer([3 3],4,”channel-wise”,”Name”,”groupedconv”,”Padding”,”same”) geluLayer(“Name”,”gelu_1”) convolution2dLayer([1 1],96,”Name”,”conv_1”,”Padding”,”same”) batchNormalizationLayer(“Name”,”batchnorm_1”)]; lgraph = addLayers(lgraph,tempLayers); tempLayers = [ additionLayer(2,”Name”,”addition”) layerNormalizationLayer(“Name”,”layernorm_1”)]; lgraph = addLayers(lgraph,tempLayers); tempLayers = [ groupedConvolution2dLayer([3 3],4,”channel-wise”,”Name”,”groupedconv_1”,”Padding”,”same”) geluLayer(“Name”,”gelu_2”) convolution2dLayer([1 1],96,”Name”,”conv_2”,”Padding”,”same”) batchNormalizationLayer(“Name”,”batchnorm_2”)]; lgraph = addLayers(lgraph,tempLayers); tempLayers = [ additionLayer(2,”Name”,”addition_1”) geluLayer(“Name”,”gelu_13”) groupedConvolution2dLayer([2 2],2,”channel-wise”,”Name”,”groupedconv_2”,”Padding”,”same”,”Stride”,[2 2]) layerNormalizationLayer(“Name”,”layernorm_2”) geluLayer(“Name”,”gelu_3”)]; lgraph = addLayers(lgraph,tempLayers); tempLayers = [ layerNormalizationLayer(“Name”,”layernorm_3”) groupedConvolution2dLayer([3 3],4,”channel-wise”,”Name”,”groupedconv_3”,”Padding”,”same”) geluLayer(“Name”,”gelu_4”) convolution2dLayer([1 1],192,”Name”,”conv_3”,”Padding”,”same”) batchNormalizationLayer(“Name”,”batchnorm_3”)]; lgraph = addLayers(lgraph,tempLayers); tempLayers = [ additionLayer(2,”Name”,”addition_2”) layerNormalizationLayer(“Name”,”layernorm_4”)]; lgraph = addLayers(lgraph,tempLayers); tempLayers = [ groupedConvolution2dLayer([3 3],4,”channel-wise”,”Name”,”groupedconv_4”,”Padding”,”same”) geluLayer(“Name”,”gelu_5”) convolution2dLayer([1 1],192,”Name”,”conv_4”,”Padding”,”same”) batchNormalizationLayer(“Name”,”batchnorm_4”)]; lgraph = addLayers(lgraph,tempLayers); tempLayers = [ additionLayer(2,”Name”,”addition_3”) geluLayer(“Name”,”gelu_12”) groupedConvolution2dLayer([2 2],2,”channel-wise”,”Name”,”groupedconv_5”,”Padding”,”same”,”Stride”,[2 2]) layerNormalizationLayer(“Name”,”layernorm_5”) geluLayer(“Name”,”gelu_6”)]; lgraph = addLayers(lgraph,tempLayers); tempLayers = [ layerNormalizationLayer(“Name”,”layernorm_6”) groupedConvolution2dLayer([3 3],4,”channel-wise”,”Name”,”groupedconv_6”,”Padding”,”same”) geluLayer(“Name”,”gelu_7”) convolution2dLayer([1 1],384,”Name”,”conv_5”,”Padding”,”same”) batchNormalizationLayer(“Name”,”batchnorm_5”)]; lgraph = addLayers(lgraph,tempLayers); tempLayers = [ additionLayer(2,”Name”,”addition_4”) layerNormalizationLayer(“Name”,”layernorm_7”)]; lgraph = addLayers(lgraph,tempLayers); tempLayers = [ groupedConvolution2dLayer([3 3],4,”channel-wise”,”Name”,”groupedconv_7”,”Padding”,”same”) geluLayer(“Name”,”gelu_8”) convolution2dLayer([1 1],384,”Name”,”conv_6”,”Padding”,”same”) batchNormalizationLayer(“Name”,”batchnorm_6”)]; lgraph = addLayers(lgraph,tempLayers); tempLayers = [ additionLayer(2,”Name”,”addition_5”) geluLayer(“Name”,”gelu_14”) groupedConvolution2dLayer([2 2],2,”channel-wise”,”Name”,”groupedconv_8”,”Padding”,”same”,”Stride”,[2 2]) layerNormalizationLayer(“Name”,”layernorm_8”) geluLayer(“Name”,”gelu_9”)]; lgraph = addLayers(lgraph,tempLayers); tempLayers = [ layerNormalizationLayer(“Name”,”layernorm_9”) groupedConvolution2dLayer([3 3],4,”channel-wise”,”Name”,”groupedconv_9”,”Padding”,”same”) geluLayer(“Name”,”gelu_10”) convolution2dLayer([1 1],768,”Name”,”conv_7”,”Padding”,”same”) batchNormalizationLayer(“Name”,”batchnorm_7”)]; lgraph = addLayers(lgraph,tempLayers); tempLayers = [ additionLayer(2,”Name”,”addition_6”) layerNormalizationLayer(“Name”,”layernorm_10”)]; lgraph = addLayers(lgraph,tempLayers); tempLayers = [ groupedConvolution2dLayer([3 3],4,”channel-wise”,”Name”,”groupedconv_10”,”Padding”,”same”) geluLayer(“Name”,”gelu_11”) convolution2dLayer([1 1],768,”Name”,”conv_8”,”Padding”,”same”) batchNormalizationLayer(“Name”,”batchnorm_8”)]; lgraph = addLayers(lgraph,tempLayers); tempLayers = [ additionLayer(2,”Name”,”addition_7”) geluLayer(“Name”,”gelu_15”) convolution2dLayer([1 1],1280,”Name”,”conv_9”,”Padding”,”same”) layerNormalizationLayer(“Name”,”layernorm_11”) geluLayer(“Name”,”gelu_16”) batchNormalizationLayer(“Name”,”batchnorm_9”) globalAveragePooling2dLayer(“Name”,”gapool”) fullyConnectedLayer(2,”Name”,”fc”) softmaxLayer(“Name”,”softmax”) classificationLayer(“Name”,”classoutput”)]; lgraph = addLayers(lgraph,tempLayers); % clean up helper variable clear tempLayers; Connect Layer Branches Connect all the branches of the network to create the network graph. lgraph = connectLayers(lgraph,”gelu”,”layernorm”); lgraph = connectLayers(lgraph,”gelu”,”addition/in2”); lgraph = connectLayers(lgraph,”batchnorm_1”,”addition/in1”); lgraph = connectLayers(lgraph,”layernorm_1”,”groupedconv_1”); lgraph = connectLayers(lgraph,”layernorm_1”,”addition_1/in1”); lgraph = connectLayers(lgraph,”batchnorm_2”,”addition_1/in2”); lgraph = connectLayers(lgraph,”gelu_3”,”layernorm_3”); lgraph = connectLayers(lgraph,”gelu_3”,”addition_2/in2”); lgraph = connectLayers(lgraph,”batchnorm_3”,”addition_2/in1”); lgraph = connectLayers(lgraph,”layernorm_4”,”groupedconv_4”); lgraph = connectLayers(lgraph,”layernorm_4”,”addition_3/in1”); lgraph = connectLayers(lgraph,”batchnorm_4”,”addition_3/in2”); lgraph = connectLayers(lgraph,”gelu_6”,”layernorm_6”); lgraph = connectLayers(lgraph,”gelu_6”,”addition_4/in2”); lgraph = connectLayers(lgraph,”batchnorm_5”,”addition_4/in1”); lgraph = connectLayers(lgraph,”layernorm_7”,”groupedconv_7”); lgraph = connectLayers(lgraph,”layernorm_7”,”addition_5/in1”); lgraph = connectLayers(lgraph,”batchnorm_6”,”addition_5/in2”); lgraph = connectLayers(lgraph,”gelu_9”,”layernorm_9”); lgraph = connectLayers(lgraph,”gelu_9”,”addition_6/in2”); lgraph = connectLayers(lgraph,”batchnorm_7”,”addition_6/in1”); lgraph = connectLayers(lgraph,”layernorm_10”,”groupedconv_10”); lgraph = connectLayers(lgraph,”layernorm_10”,”addition_7/in1”); lgraph = connectLayers(lgraph,”batchnorm_8”,”addition_7/in2”); Plot Layers plot(lgraph);

**Table A2:** Python code of the proposed GPTNeXt. (Python 3.9+; TensorFlow 2.12.0+; NumPy 1.23.0+).

from tensorflow.keras.models import Model from tensorflow.keras.layers import Input, Conv2D, BatchNormalization, LayerNormalization, Add, Activation, GlobalAveragePooling2D, Dense, Softmax # Create Layer Graph lgraph = None # Add Layer Branches input_layer = Input(shape = (224, 224, 3), name = “imageinput”) x = Conv2D(96, kernel_size = (4, 4), padding = “same”, strides = (4, 4), name = “conv”)(input_layer) x = BatchNormalization(name = “batchnorm”)(x) x = Activation(“gelu”, name = “gelu”)(x) # Create the first branch branch1 = Model(inputs = input_layer, outputs = x) # Create the second branch input_layer = branch1.output x = LayerNormalization(name = “layernorm”)(input_layer) x = Conv2D(4, kernel_size = (3, 3), padding = “same”, name = “groupedconv”, strides = (1, 1), activation = “gelu”)(x) x = Conv2D(96, kernel_size = (1, 1), padding = “same”, name = “conv_1”)(x) x = BatchNormalization(name = “batchnorm_1”)(x) x = Activation(“gelu”, name = “gelu_1”)(x) branch2 = Model(inputs = branch1.input, outputs = x) # Create the third branch input_layer = branch2.output x = Add(name = “addition”) ([input_layer, branch1.output]) x = LayerNormalization(name = “layernorm_1”)(x) branch3 = Model(inputs = branch2.input, outputs = x) # Repeat the process for the remaining branches... # Connect Layer Branches lgraph = branch1 lgraph = Model(inputs = lgraph.input, outputs = branch3(lgraph.output)) lgraph = Model(inputs = lgraph.input, outputs = branch2(lgraph.output)) lgraph = Model(inputs = lgraph.input, outputs = branch1(lgraph.output))
